# Electrolyte and acid-base disorders in cancer patients and its impact on clinical outcomes: evidence from a real-world study in China

**DOI:** 10.1080/0886022X.2020.1735417

**Published:** 2020-03-05

**Authors:** Yang Li, Xiaohong Chen, Ziyan Shen, Yimei Wang, Jiachang Hu, Jiarui Xu, Bo Shen, Xiaoqiang Ding

**Affiliations:** a Department of Nephrology, Zhongshan Hospital, Fudan University, Shanghai, China; b Shanghai Medical Center of Kidney, Shanghai, China; c Shanghai Key Laboratory of Kidney and Blood Purification, Shanghai, China; d Shanghai Institute of Kidney and Dialysis, Shanghai, China; e Hemodialysis Quality Control Center of Shanghai, Shanghai, China

**Keywords:** Cancer, electrolyte, acid-base, clinical epidemiology

## Abstract

**Background:**

This study aims to delineate the incidence of electrolyte and acid-base disorders (EAD) in cancer patients, to figure out the risk factors of EAD, then to assess the impact of EAD on patients’ in-hospital clinical outcomes.

**Methods:**

Patients with the diagnosis of malignancies hospitalized during 1 October 2014 and 30 September 2015 were recruited in Zhongshan Hospital, Fudan University in Shanghai of China. Demographic characteristics, comorbidities, and clinical data, including survival, length of stay and hospital cost, were extracted from the electronic medical record system. Electrolyte and acid-base data were acquired from the hospital laboratory database.

**Results:**

Of 25,881 cancer patients with electrolyte data, 15,000 (58.0%) cases had at least one electrolyte and acid-base abnormity. Hypocalcemia (27.8%) was the most common electrolyte disorder, followed by hypophosphatemia (26.7%), hypochloremia (24.5%) and hyponatremia (22.5%). The incidence of simple metabolic acidosis (MAC) and metabolic alkalosis (MAL) was 12.8% and 22.1% respectively. Patients with mixed metabolic acid-base disorders (MAC + MAL) accounted for 30.2%. Lower BMI score, preexisting hypertension and diabetes, renal dysfunction, receiving surgery/chemotherapy, anemia and hypoalbuminemia were screened out as the major risk factors of EAD. In-hospital mortality in patients with EAD was 2.1% as compared to those with normal electrolytes (0.3%). The risk of death significantly increased among patients with severe EAD. Similarly, the length of stay and hospital cost also tripled as the number and grade of EAD increased.

**Conclusion:**

EAD is commonly encountered in cancer patients and associated with an ominous prognosis. Patients with comorbidities, renal/liver dysfunction, and anti-tumor therapy have a higher risk of EAD. Regular monitoring of electrolytes, optimum regimen for intravenous infusion, timely correction of modifiable factors and appropriate management of EAD should not be neglected during anti-tumor treatment.

## Introduction

Cancer is rapidly emerging as an important cause of mortality and morbidity globally in recent years [1]. In 2016, there were 17.2 million cancer cases worldwide and 8.9 million deaths. The global cancer cases increased by 28% over the past decade [2]. As one of the largest developing countries, new diagnosed cancer cases in China in 2015 reached 4.2 million with an incidence of 201.1 per 100,000 [3].

Electrolyte and acid-base disorders (EAD) are the prelude of the disequilibrium of the human body, involving mechanisms of malnutrition, organ decompensate, endocrine dyscrasia, etc. It makes EAD ubiquitous in patients diagnosed with cancer. Previous studies reported the incidence of hyponatremia and hypokalemia in cancer patients was up to 47–64% [4,5] and 41–48% [6,7] respectively. The occurrence of various EAD is concomitant and can trigger a series of symptoms such as delirium, fatigue, constipation, nausea, vomiting, and even the in-hospital death [8,9]. In most cases, EAD is associated with the etiology seen in general inpatients and not specifically allied to the underlying cancer. While in others, it is caused by anti-tumor treatment or due to the existence of paraneoplastic syndromes [10]. It is estimated that the direct cost of treating hyponatremia in the United States reached $1.6∼$3.6 billion annually [11], let alone treating other types of EAD with far more significant consequences.

Early detection and prompt correction of EAD can improve patients’ short-term outcome and quality of life [12]. However, the epidemiology of EAD in cancer patients and its relationship with clinical outcomes remains to be studied. To this end, our study aims to delineate the incidence of EAD in patients with different malignancies, to identify the factors that could increase the risks of EAD, then to assess the impact of EAD on patients’ in-hospital mortality and healthcare utilization.

## Materials and methods

### Study design and population

This study was designed as a real-world, retrospective cohort study based on the electronic medical records database in Zhongshan Hospital, Fudan University in Shanghai of China. Patients who were diagnosed with cancer admitted into the hospital between 1 October 2014 and 30 September 2015 were recruited. The exclusion criteria included the following: (1) patients with a stay of admission less than 24 hours; (2) patients under 14 years old; (3) patients on maintenance dialysis; (4) patients with renal transplantation and (5) patients without electrolyte or acid-base tests. If a patient was admitted multiple times during the study period, we regarded each hospitalization as an independent case. Ethics clearance was issued by the institutional review board of Zhongshan Hospital, Fudan University (No.B2016-103).

### Data collection

Demographic information, comorbidities, clinical records, as well as electrolyte and acid-base records were acquired from the hospital electronic medical records (EMR) databases. Before data extraction, personal identity information was replaced with codes for privacy concerns. Renal function was measured by the estimated glomerular filtration rate (eGFR) and serum uric acid (SUA). The level of eGFR was calculated by using the chronic kidney disease epidemiology collaboration (CKD-EPI) 2009 creatinine equation [13]. Liver function was measured by aspartate aminotransferase (AST) and alanine aminotransferase (ALT). Other biochemical indicators contained albumin, hemoglobin, and leukocyte count. In-hospital mortality was regarded as the primary clinical outcome, and the secondary outcomes were length of stay and hospital cost.

### Definition of electrolyte and acid-base disorders

Electrolytes for analysis include serum sodium, potassium, chloride, calcium, magnesium, and phosphorus. The reference value range is 137–147 mmol/L in sodium, 3.5–5.3 mmol/L in potassium, 99–110 mmol/L in chloride, 2.15–2.55 mmol/L in calcium, 0.67–1.04 mmol/L in magnesium and 0.90–1.34 mmol/L in phosphorus respectively. A value less than the lower range of each electrolyte is considered as hypo-electrolytemia, and a value greater than the upper range is considered as hyper-electrolytemia. Arterial blood gas values were measured by Medica Easy Electrolytes (Medica Corporation, Bedford, MA, USA). Acidemia is regarded as arterial blood gas with a pH value less than 7.35, while alkalemia refers to that with a pH value higher than 7.45. The anion gap (AG) was calculated by the formula [14]: AG = [Na^+^] − [Cl^−^] − [HCO_3_^−^], with an elevated AG of greater than 16 mmol/L. Simple, dual, and triple acid-base disorders were diagnosed based on the level of pH, HCO_3_^−^, anion gap (AG), and pCO2 through the flow diagrams developed by Fulop M [15].

An EAD ‘event’ means that patient suffered from at least one kind of electrolyte or acid-base disorders during the hospitalization. The incidence of EAD is calculated as the number of EAD cases divided by patients receiving electrolyte tests. According to the National Cancer Institute Common Terminology Criteria for Adverse Events, Version 5.0 (NCI-CTCAE. V5.0) [16], the severity of EAD is ranked from grade 1 to grade 4 (Supplementary Table 1). Considering the synergistic effect of EAD, the severity upgrades correspondingly when patients co-occurred two or more abnormal electrolytes or acid-base disorders.

### Diagnosis of cancer and acute kidney injury

Cancer diagnosis was categorized into 28 subgroups according to the International Classification of Diseases, 11th Revision (ICD-10) [17]. The presence of comorbidities was determined by the diagnosis records at admission and discharge. Anti-tumor treatment was classified into surgery, chemotherapy, interventional therapy, and untreated/palliative care. Acute kidney injury (AKI) was defined as an absolute increase in serum creatinine (SCr) by ≥0.3 mg/dl (26·5 μmol/L) within 48 h or ≥1.5-fold from the baseline within the previous 7 days in hospitalization [18].

### Statistical analysis

Continuous variables are described as mean ± standard deviation or median with interquartile range, whereas categorical variables are presented as numbers and percentages. Epidemiological distribution of EAD is figured with histogram and pie graph. After adjusting the demographic factors, the adjusted odds ratio (aOR) and its 95% confidence interval (CI) of associated factors with AKI are estimated, including present comorbidity, treatment, renal/liver function, and biochemical test. Heat map is applied to delineate the specific EAD incidence in different cancer sites. Survival probability and median survival time are used to illustrate in-hospital mortality in varied EAD counts and grades. The adjusted hazard ratio (aHR) is calculated in cox proportional hazard model to identify the predicting factors with mortality. Box plots are applied to describe hospital cost and length of stay among patients with different EAD count and grade. Significant level (α) is set at 0.05. All data analysis was run using SAS v9.4 software (SAS Institute, Cary, North Carolina) and R v3.6.0 software (R core team, https://www.r-project.org).

## Results

**Figure 1.**
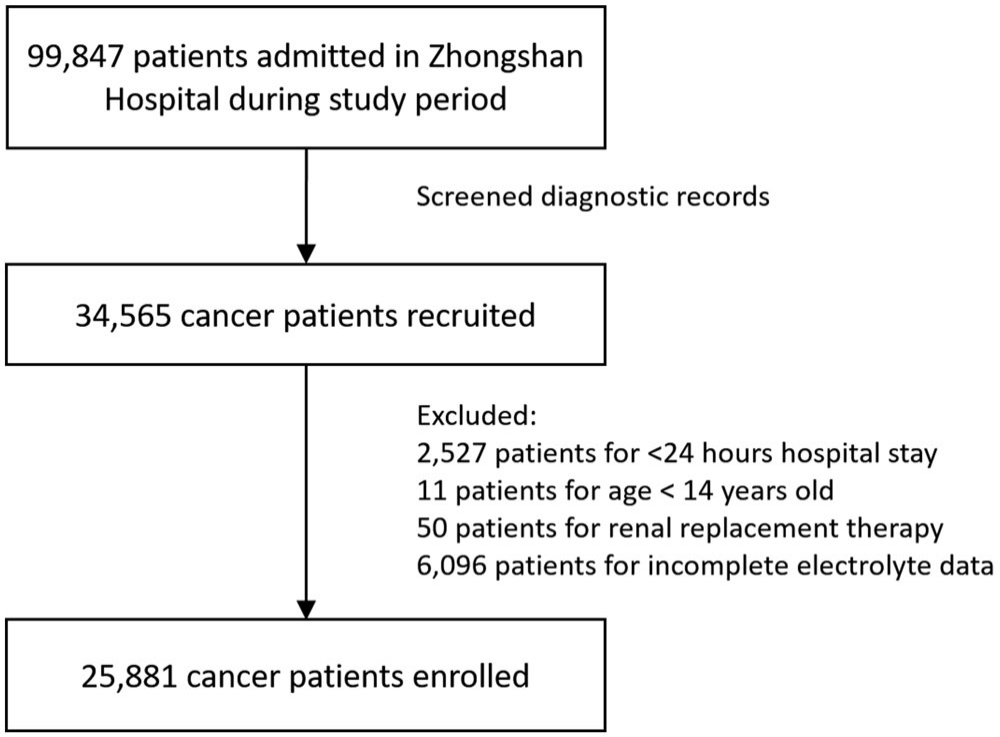
Flow chart of the study population selection.

Of the 34,565 admissions with malignancy recruited by retrieving medical records, 25,881 admissions were enrolled in the study (Figure 1). The average age of patients was 58.8 ± 12.6 years old and the male accounted for 66.6%.

### Incidence of electrolyte and acid-base disorders

**Figure 2.**
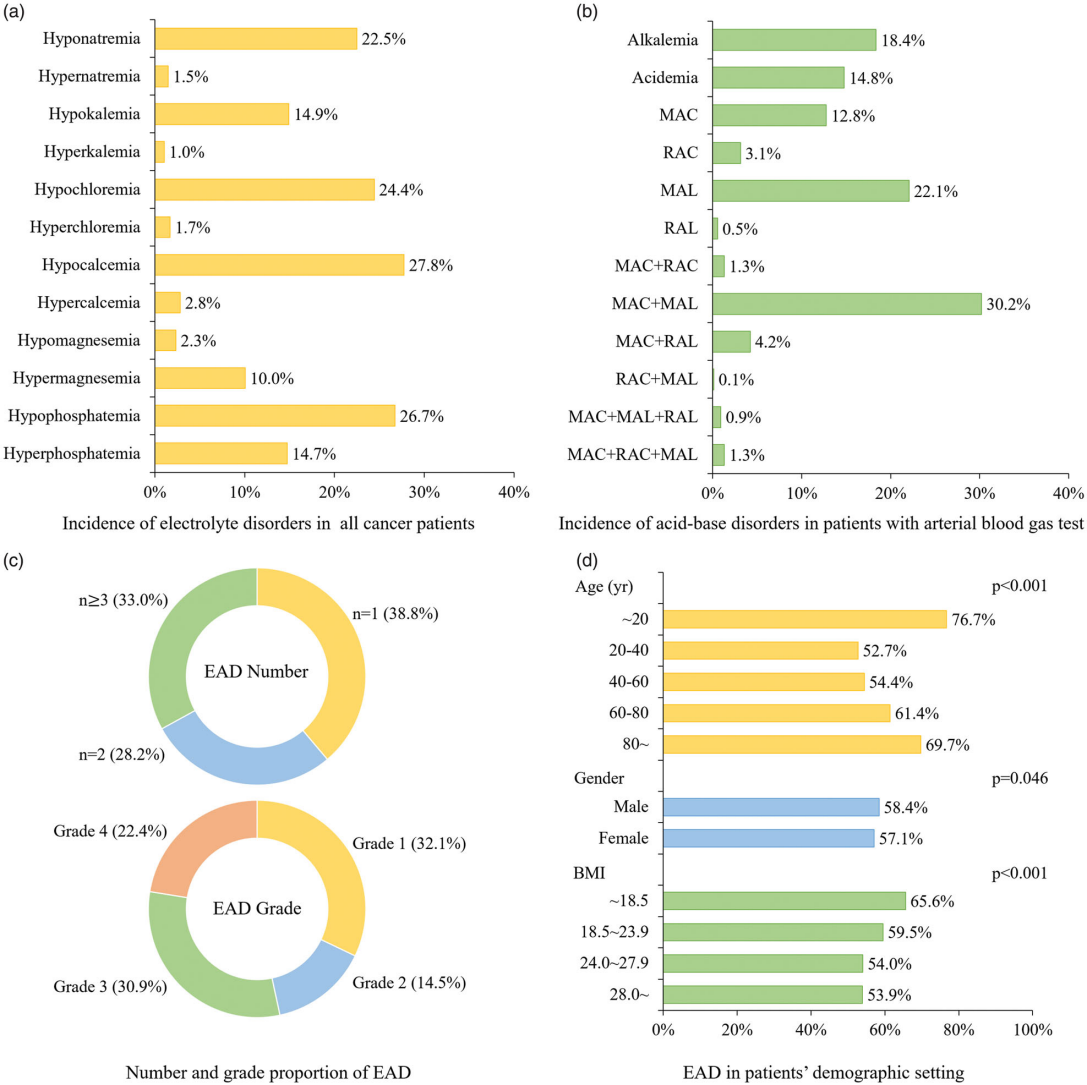
Epidemiological distribution of electrolyte and acid-base disorders in cancer patients. (a) The incidence of electrolyte disorders was presented on the bar graph in the all cancer patients. (b) The incidence of acid-base disorders was presented on the bar graph in patients with arterial blood gas test. (c) The number and grade of EAD was presented on the annular chart. (d) The incidence of EAD was classified in varied demographic factors and presented on the bar graph with labels. *EAD: electrolyte and acid-base disorders; MAC: metabolic acidosis; MAL: metabolic alkalosis; RAC: Respiratory acidosis; RAL: respiratory alkalosis*.

All of the eligible participants had received sodium, potassium and chloride tests. Of them, about two-thirds measured the level of magnesium and phosphorus (*n* = 16,940). Acid-base level was measured in 3,484 patients (13.5%) in the format of arterial blood gas analysis who were usually in critical status. Totally 15,000 patients (58.0%) were recognized with at least one category of electrolyte and acid-base abnormalities. Hypocalcemia (27.8%) was the most common electrolyte disorder (Figure 2(a)), followed by hypophosphatemia (26.7%), hypochloremia (24.4%) and hyponatremia (22.5%). The incidence of simple metabolic acidosis (MAC) and metabolic alkalosis (MAL) was 12.8% and 22.1% respectively. Patients with dual metabolic acid-base disorders (MAC + MAL) accounted for 30.2% (Figure 2(b)).

**Figure 3.**
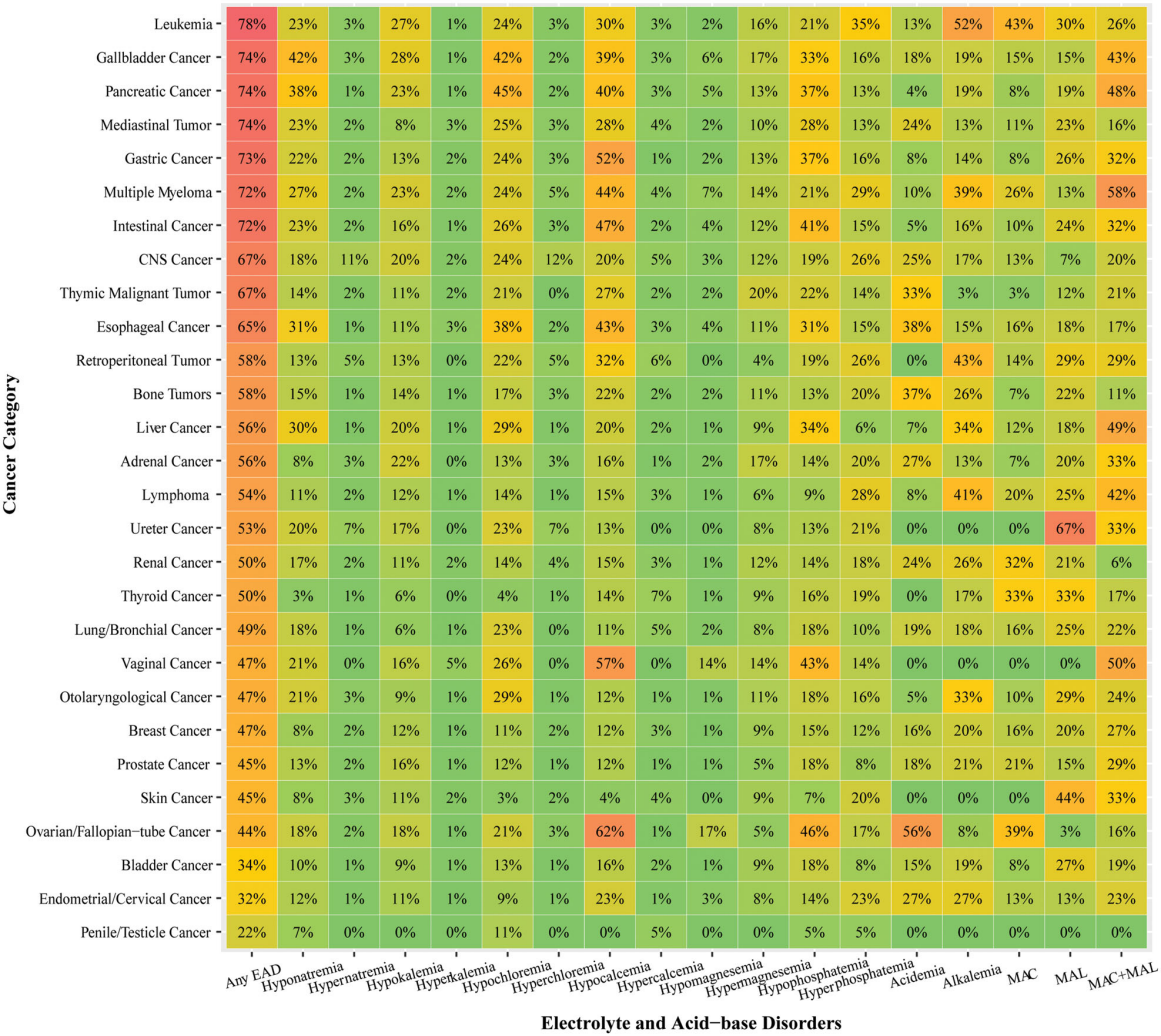
Heat map of EAD in patients with varied cancer sites. *EAD: electrolyte and acid-base disorders*.

As shown in Figure 2(c), one-third of EAD patients had over three categories of EAD. Over half of patients were in EAD grade 3 and grade 4. In terms of demographic factors, a ‘U’ shape trend was observed in age-specific EAD incidence: either patients aged <20 years or ≥80 years were more vulnerable to EAD (Figure 2(d)). Yet the EAD incidence did not vary notably with gender. Patients with lower BMI (<18.5) shared the highest EAD incidence (65.6%). Heat map revealed that EAD incidence varied in different cancer sites (Figure 3). Patients with leukemia (78%), gallbladder cancer (74%) and pancreatic cancer (74%) had the highest incidence of EAD. In particular, liver cancer cases shared a higher incidence of hyponatremia (30%) and MAC + MAL (49%), while the highest incidence of hypocalcemia and hypophosphatemia occurred in patients with female reproductive malignancy. The top 5 EAD for different cancer categories were listed in Supplementary Figure 1.

### Risk factors for EAD incidence

As shown in Table 1, in-hospital AKI was observed as the major risk factors contributing to EAD (aOR = 3.09). Preexisting hypertension and diabetes were also associated with a higher EAD incidence (aOR = 1.14/1.14). Patients with poor eGFR (<60 mL/min/1.73m^2^) and severe hyperuricemia (≥540 μmol/L) showed a higher probability of EAD (aOR = 1.41/2.12). Compared with untreated/palliative care, patients receiving surgery (aOR: 5.61), chemotherapy (aOR = 1.55) and interventional therapy (aOR = 1.18) were more predisposed to EAD. Another positive association with EAD incidence was observed in abnormal biochemical test. The aORs of patients with lower albumin and hemoglobin level were estimated as 2.64 and 1.67, respectively. No significant difference was found when investigating into cancer category and stage.

**Table 1.** Clinical factors for electrolyte and acid-base disorders (EAD) incidence and in-hospital mortality.

	EAD incidence (*n* = 25,881)			In-hospital mortality (*n* = 15,000)		
	Total	Yes, n (%)	aOR (95% CI)^a^	Total	Yes, n (%)	aHR (95% CI)^b^
Comorbidities						
Hypertension	3722	2286 (61.4)	1.14 (1.06∼1.22)	2286	73 (3.2)	0.93 (0.70∼1.23)
Diabetes	1919	1188 (61.9)	1.14 (1.04∼1.26)	1188	41 (3.5)	1.52 (1.07∼2.15)
AKI	3264	2598 (79.6)	3.09 (2.82∼3.38)	2598	167 (6.4)	1.72 (1.37∼2.18)
Renal/liver function						
eGFR (<60 mL/min/1.73m^2^)	1444	981 (67.9)	1.41 (1.25∼1.58)	981	70 (7.1)	2.11 (1.58∼2.83)
SUA (≥540 μmol/L)	434	325 (74.9)	2.12 (1.70∼2.63)	325	44 (13.5)	3.94 (2.80∼5.55)
ALT (<80 U/L)	1383	986 (71.3)	1.90 (1.68∼2.14)	986	54 (5.5)	2.99 (2.22∼4.04)
AST (<70 U/L)	2234	1634 (73.1)	2.09 (1.90∼2.31)	1634	92 (5.6)	4.42 (3.43∼5.68)
Treatment						
Surgery	6200	4956 (79.9)	5.61 (4.94∼6.36)	4956	10 (0.2)	0.13 (0.06∼0.28)
Chemotherapy	11795	6347 (53.8)	1.55 (1.38∼1.74)	6347	240 (3.8)	2.78 (1.64∼4.72)
Interventional therapy	6434	3041 (47.3)	1.18 (1.05∼1.33)	3041	19 (0.6)	1.41 (0.73∼2.70)
Untreated/palliative care	1452	656 (45.2)	1.00	656	39 (5.9)	1.00
Cancer category						
Non-hematologic	23507	13628 (58.0)	1.00	13628	238 (1.7)	1.00
Hematologic	2374	1372 (57.8)	1.01 (0.93∼1.11)	1372	70 (5.1)	1.66 (1.25∼2.22)
Cancer Stage						
Loco-regional	23892	13815 (57.8)	1.00	13578	252 (1.8)	1.00
Distant metastasis	1989	1185 (59.6)	1.07 (0.97∼1.17)	1182	56 (4.7)	2.71 (2.00∼3.66)
Biochemical test						
Albumin (<35 g/L)	4659	3578 (76.8)	2.64 (2.46∼2.85)	3578	197 (5.5)	3.77 (2.94∼4.83)
Hemoglobin (<115 g/L)	7891	5289 (67.0)	1.67 (1.57∼1.77)	5289	201 (3.8)	1.96 (1.54∼2.50)
Leukocyte (≥9.5 × 10^9^)	1969	1505 (76.4)	2.46 (2.21∼2.74)	1505	121 (8.0)	3.72 (2.94∼4.72)

EAD: electrolyte and acid-base disorders; aOR: adjusted odds ratio; aHR: adjusted hazard ratio; AKI: acute kidney injury; eGFR: estimated glomerular filtration rate; SUA: serum uric acid; ALT: alanine aminotransferase; AST: aspartate aminotransferase.

^a^ The odds ratio (OR) and its 95% CI was calculated in multivariate logistic regression after adjusting the demographic factors including age, gender, residence and BMI.

^b^ The hazard ratio (HR) and its 95% CI was calculated in cox proportional hazard model after adjusting the demographic factors including age, gender, residence and BMI.

Assuming the renal/liver function and biochemical results are remediable in advance, we regarded these features as modifiable factors for the progression of EAD. Table 2 showed the joint effect of these factors and anti-tumor treatment on EAD. The risk of EAD among patients with modifiable factors significantly increased in different treatment settings with an aOR ranged from 1.67 to 3.00. It implied that correcting these modifiable factors on the initial anti-tumor treatment could decrease the likelihood of EAD remarkably.

**Table 2.** Joint effect of modifiable factors and anti-tumor treatment on electrolyte and acid-base disorders.

Treatment	Modifiable factors^a^	EAD incidence	OR (95% CI)	*p* Value
Surgery	Yes	1830 (85.7)	1.80 (1.56∼2.07)	<0.001
	No	3126 (76.9)	1.00	
Chemotherapy	Yes	4158 (66.3)	3.00 (2.78∼3.23)	<0.001
	No	2189 (39.6)	1.00	
Interventional therapy	Yes	1619 (54.0)	1.67 (1.51∼1.84)	<0.001
	No	1422 (41.4)	1.00	
Untreated/palliative care	Yes	366 (59.3)	2.74 (2.21∼3.40)	<0.001
	No	290 (34.7)	1.00	

eGFR: estimated glomerular filtration rate; SUA: serum uric acid; ALT: alanine aminotransferase; AST: aspartate aminotransferase; EAD: electrolyte and acid-base disorders.

^a^ Modifiable factors were defined as those who had abnormal renal/liver function or biochemical tests (including eGFR, SUA, ALT, AST, album, hemoglobin and leukocyte).

### In-hospital mortality among EAD patients

**Figure 4.**
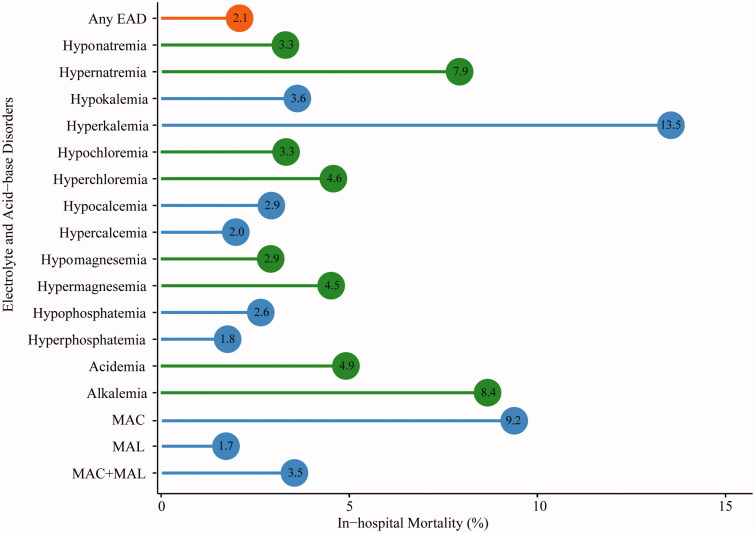
In-hospital mortality among patients with electrolyte and acid-base disorders. *MAC: metabolic acidosis; MAL: metabolic alkalosis*.

**Figure 5.**
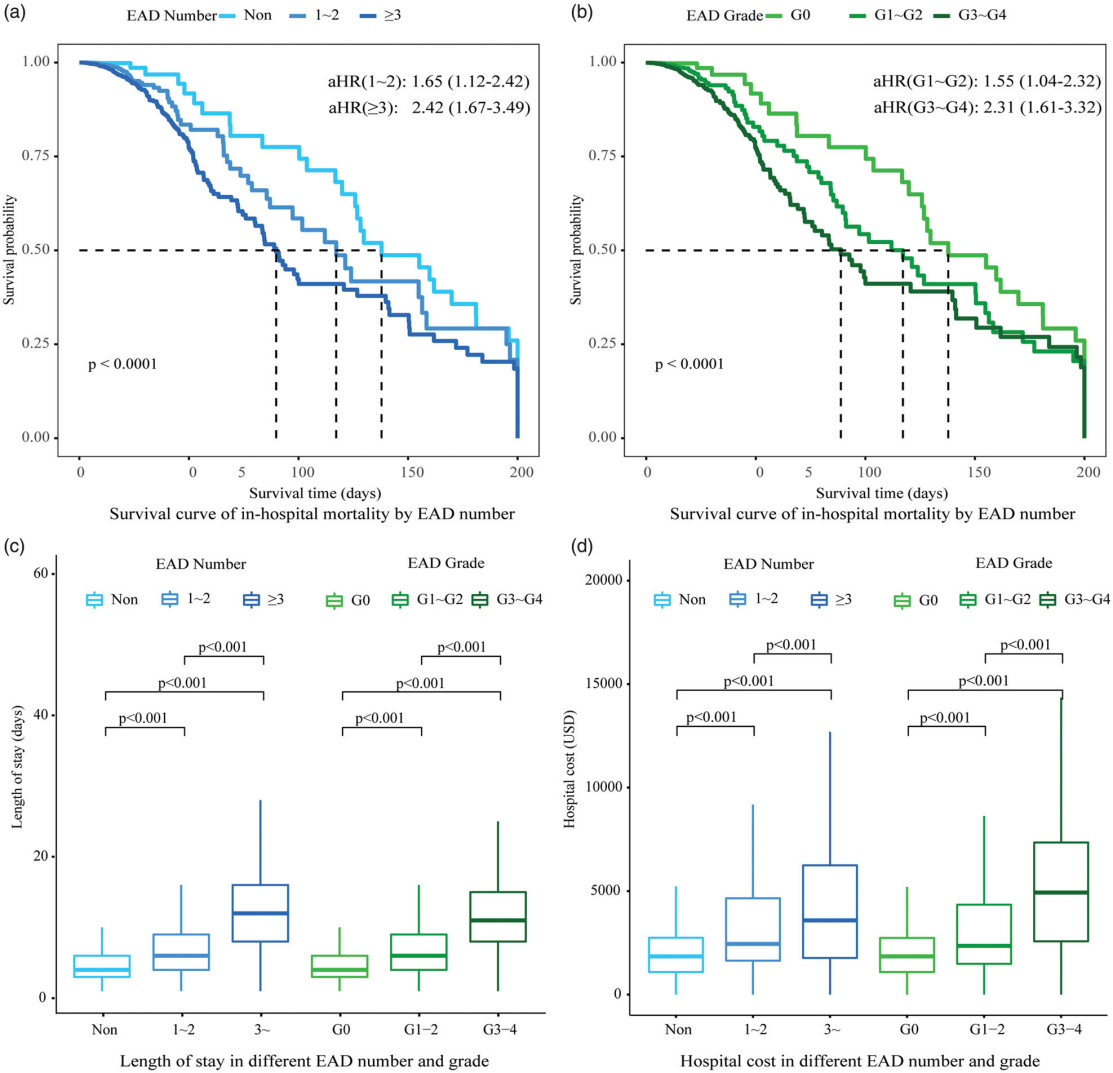
Outcomes of cancer patients with electrolyte and acid-base disorders. (a) The in-hospital mortality was presented in the survival curve and classified by EAD number (b) The in-hospital mortality was presented in the survival curve and classified by EAD Grade. (c) The length of stay was presented on the boxplot. Kruskal–Wallis test was applied to identify the differences of length of stay classified by EAD number and grade. (d) Hospital cost was presented on the boxplot. Kruskal–Wallis test was applied to identify the differences of hospital cost classified by EAD number and grade. *EAD: electrolyte and acid-base disorders; 1 USA dollar ≈ 6.96 China Yuan*.

In cancer patients with EAD, the in-hospital mortality rate was 2.1%, which was significantly higher than those with normal electrolytes (0.3%, HR = 2.00, 95%CI 1.42∼2.83). The top EAD associated with high mortality were as follows: hyperkalemia (13.5%), MAC (9.2%), alkalemia (8.4%) and hypernatremia (7.9%; Figure 4). In survival analysis, a gradient increase in the risk of death was found along with the number of EAD and EAD severity (Figure 5(a,b). Compared with non-EAD patients, the median survival time in cases with over three kinds of EAD was much shorter (89 days vs. 138 days, *p* < 0.001) with an adjusted HR of 2.42. Similarly, patients with grade 3–4 EAD had a higher risk of in-hospital mortality (aHR = 2.31) than patients without EAD.

### Length of stay and hospital cost

Healthcare utilization associated with EAD during hospitalization was illustrated in Figure 5(c,d). With the increase of EAD number, the median length of stay significantly increased from 4.0 days to 11.5 days and so did the hospital cost (from $1910 to $5382). With regard to the EAD severity, the length of stay ranked from high to low was grade 3–4 (8.0 days), grade 1–2 (7.0 days) and non-EAD (4.0 days). Similarly, the highest hospital cost was also observed in grade 3–4 EAD patients ($3823), which was significantly higher than grade 1–2 EAD patients ($2557, *p* < 0.001) and non-EAD patients ($1910, *p* < 0.001).

## Discussion

In this study, the incidence of EAD was estimated as 58.0% in cancer patients, which was remarkably higher than reported populations, including patients referred to the emergency department (13.7%) [19] and the elderly (22.0%) [20]. It suggests that EAD in cancer patients should be paid as much attention as other medical conditions, especially in those receiving surgery and chemotherapy. Stratified by cancer sites, patients with leukemia (78%) had the highest incidence of EAD. Another study in fifty-four patients with acute leukemia also reported that 75.9% had at least one EAD disorders [21]. Leukemia-related electrolyte disorders are mainly considered to be associated with the leukemic process, organ infiltration, cell death and/or therapeutic interventions. Besides, we found that acid-base disorder was encountered in most of the leukemia patients (MAC 43%, MAL 30%, MAC + MAL 26%). MAC is probably related to obstinate vomiting with increased upper gastrointestinal losses induced by chemotherapy, resulting in hypovolemia and hypokalemia [22]. MAL is due to the presence of renal failure [23].

It was observed that 22.5% and 14.9% of cancer patients suffered from hyponatremia and hypokalemia respectively in this study. Volume depletion is the main reason for hyponatremia, which usually occurred in hemorrhage, diarrhea, vomiting, drainage of ascites or pleural effusion, peritonitis, or ileus [24]. Besides, syndrome of inappropriate antidiuretic hormone (SIADH) is considered as another common cause of hyponatremia in cancer condition. Sorensen et al. reviewed that SIADH occurs in 3% of patients with head and neck cancer, in 0.7% of patients with non-small cell lung cancer, and in 15% of patients with small cell lung cancer [25]. In terms of the hypokalemia mechanism, poor nutrition, anorexia and volume depletion can induce inadequate potassium intake. Vomiting generates the loss of potassium. Alkalemia also causes a shift of potassium into cells, thus producing hypokalemia. Furthermore, in patients with leukemia, the increased production of blast cells can lead to hypokalemia [26]. Besides, we found that hypocalcemia (27.8%) and hypophosphatemia (26.7%) were commonly encountered. The occurrence of hypocalcemia may be resulted from malnutrition, hypoalbuminemia, sepsis or tumor lysis syndrome (TLS) [27,28]. The release of phosphorus from damaged cells and cellular shift from intracellular to extracellular give rise to hyperphosphatemia, in situations including rhabdomyolysis, TLS, respiratory alkalosis and lactic and ketoacidosis [29]. Additionally, many of the fluids used for hydration and resuscitation contains high concentrations of chloride, and it also may induce or exacerbate hyperchloremia and MAC [30,31].

In our study, patients with chronic diseases and renal/liver dysfunction were more predisposed to EAD. Previous studies also reported that a higher EAD incidence in patients with hypertension [32], heart failure [33] and renal insufficiency [34]. Diabetes is described as an independent risk factor for hyponatremia. For every 5 mmol/l rise in serum glucose level, a decline in serum sodium by 1.6–2.4 mmol/l is reported, as glucose acts together with sodium in maintaining osmolality [35]. It suggests that EAD might be prevented by early correction of renal/liver dysfunction and other biochemical features at admission.

Anti-tumor chemotherapy, including cytotoxic drug, antibiotics and contrast agents, represent the well-established causes of EAD in cancer patients. In this study, we found that patients receiving chemotherapy and interventional therapy had a higher risk of EAD (aOR = 1.55/1.18). Cyclophosphamide, vincristine, and polymyxin B have been proved to impair renal excretion of water. Cyclophosphamide can potentiate the renal effect of anti-diuretic hormone or directly affect the tubular function, resulting in hyponatremia [24]. Polymyxin B-induced nephrotoxicity is associated with hyponatremia, hypocalcemia, and hypokalemia. The synergistic effect of anti-neoplastic drugs and electrolyte disorders may even result in fatal arrhythmias [23]. For these reasons, oncologist and nephrologist should be vigilant for the use of anti-tumor agent as well as correction of these modifiable factors of EAD before the initiation of chemotherapy as well as during treatment.

In-hospital EAD and AKI usually happen simultaneously and reinforced each other in cancer patients [34]. The target-controlled regimen for intravenous infusion can be used to identify the incidence of both EAD and AKI at an early stage. A pre- and post-control study conducted in the ICU ward found that chloride-restrictive intravenous strategy not only prevented the occurrence of hyperchloremia and MAC but decreased the incidence of the more severe stages of AKI and the use of RRT [36]. When AKI occurs, timely correction of EAD also could alleviate and even reverse AKI progression. One national report in the UK stated that in-hospital AKI could have been avoided in one-fifth of the patients if they had received better monitoring of electrolytes, recognition of risk factors, and prompt management [37].

In our study, EAD was associated with a 2.0-fold increase in the hazard ratio of in-hospital death, as well as longer length of stay and higher hospital costs, consistent with other studies [38–40]. The median survival time sustained declining along with the rise of EAD number and severity. One meta-analysis also reveals that hyponatremia patients had a higher risk of re-admission after the first hospitalization [41]. Moreover, cox regression found that not only the comorbidities of diabetes and AKI but also the cancer-related characteristics, such as chemotherapy, hematologic malignancies or cancer with distant metastasis, could further aggravate the risk of death in patients with EAD. Hence, regular monitoring of electrolytes can improve the clinical outcomes in cancer patients, especially those with advanced age, lower BMI score, underlying diseases or patients receiving surgical/chemotherapy.

We retrospectively delineate the incidence of cancer-related EAD and its associated clinical factors in a large-scale in-hospital population. Strict inclusion criteria ensured the data integrity and rationality for analysis. Notably, we described the electrolyte disorders in various clinical settings and evaluated their impacts on patients’ in-hospital mortality and healthcare utilization. However, the study was limited on certain aspects. Firstly, this was a single center-based study, so the extrapolation of research finding was restricted. Secondly, we did not collect the detailed medication history and it would obscure the relations between chemotherapy and EAD. Thirdly, we only listed the incidence of MAC, MAL and MAC + MAL in specific cancer categories due to the limited sample size in other acid-base disorder conditions. In the future, a multi-center investigation with regular follow-up after discharge is intended to be conducted to achieve a larger sample size and assess the long-term impacts of EAD.

In conclusion, electrolyte and acid-base disorders are commonly encountered in cancer patients. Patients with comorbidities, renal/liver dysfunction, and anti-tumor therapy have a higher risk of EAD, which may aggravate an ominous clinical prognosis. Regular monitoring of electrolytes, optimum regimen for intravenous infusion, correction of modifiable factors and appropriate management of EAD should not be neglected during anti-tumor treatment. Short-term outcomes and quality of life might be improved as a result.

## Supplementary Material

Supplemental Material

Supplemental Material
