# Supplementary material for: Electrolyte and acid-base disorders in cancer patients and its impact on clinical outcomes: evidence from a real-world study in China
Source: Ren Fail. 2020 Mar 5;42(1):234–43. doi: 10.1080/0886022X.2020.1735417 (PMC7067195; doi:10.1080/0886022X.2020.1735417)
Supplement: Supplemental Material [file IRNF_A_1735417_SM0548.pdf]

Supplementary Table 1. The severity classification of electrolyte and acid-base disorders

EAD	Grade 1	Grade 2	Grade 3	Grade 4
Hyponatremia	130~137 mmol/L	125~129 mmol/L	120~124 mmol/L	<120 mmol/L
Hypernatremia	147~149 mmol/L	150~154 mmol/L	155~160 mmol/L	>160 mmol/L
Hypokalemia	3.0~3.5 mmol/L	——	2.5~2.9 mmol/L	<2.5 mmol/L
Hyperkalemia	4.5~5.4 mmol/L	5.5~5.9 mmol/L	6.0~7.0 mmol/L	>7.0 mmol/L
Hypochloremia	95~99 mmol/L	90~94 mmol/L	85~89 mmol/L	<84 mmol/L
Hyperchloremia	110~114 mmol/L	115~119 mmol/L	120~125 mmol/L	>125 mmol/L
Hypocalcemia	2.0~2.15 mmol/L	1.75~1.9 mmol/L	1.5~1.74 mmol/L	<1.5 mmol/L
Hypercalcemia	2.55~2.9 mmol/L	3.0~3.1 mmol/L	3.2~3.4 mmol/L	>3.4 mmol/L
Hypomagnesemia	0.5~0.69 mmol/L	0.5~0.4 mmol/L	0.4~0.3 mmol/L	<0.3 mmol/L
Hypermagnesemia	1.04~1.23 mmol/L	——	1.23~3.30 mmol/L	>3.30 mmol/L
Hypophosphatemia	0.8~0.9 mmol/L	0.6~079 mmol/L	0.4~0.59 mmol/L	<0.4 mmol/L
Hyperphosphatemia	1.34~1.49 mmol/L	1.50~1.59 mmol/L	1.60~1.80 mmol/L	>1.80 mmol/L
Acidemia	7.3~7.35	——	<7.3	——
Alkalemia	7.45~7.5	——	>7.5	——

EAD: electrolyte and acid-base disorders.
