# Supplementary material for: Electrolyte and acid-base disorders in cancer patients and its impact on clinical outcomes: evidence from a real-world study in China
Source: Ren Fail. 2020 Mar 5;42(1):234–43. doi: 10.1080/0886022X.2020.1735417 (PMC7067195; doi:10.1080/0886022X.2020.1735417)
Supplement: Supplemental Material [file IRNF_A_1735417_SM0488.pdf]

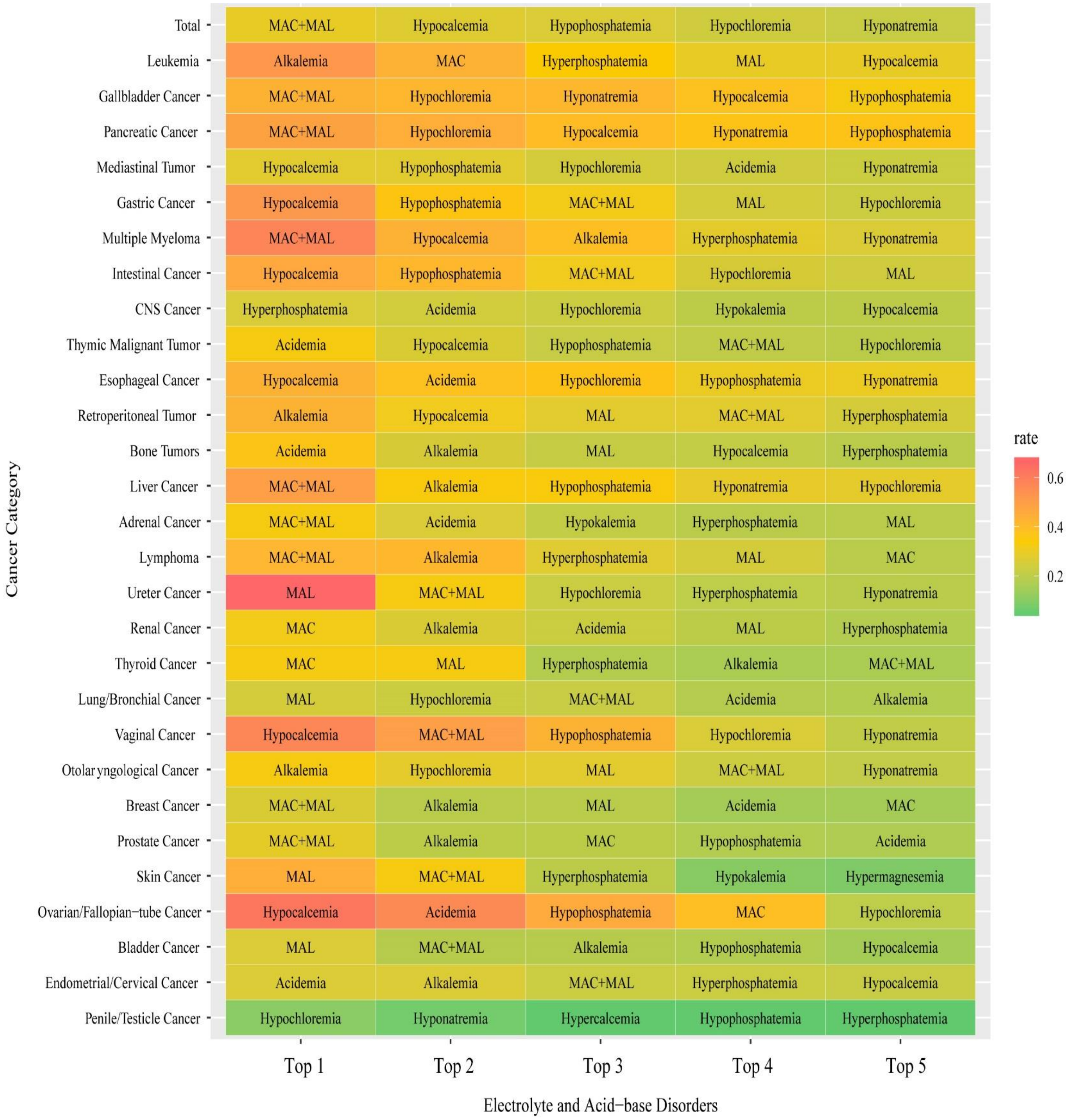

Supplementary Figure 1. The top 5 EAD for different cancer categories. *MAC: metabolic acidosis; MAL: metabolic alkalosis.*
